# A Note of Thanks to Our Reviewers

**DOI:** 10.1080/15604280214486

**Published:** 2002


					The Editors wish to extend their heartfelt
thanks to the reviewers of papers published in
the first two volumes of the Journal. Critical
but fair reviews by experts in the various fields
of diabetes research has and will be pivotal to
the quality and success of the journal. It is a
time consuming exercise in colleague's often
busy routine that they volunteer. We are there-
fore thankful for their time and expertise that
have, in no small measures, contributed to two
successful initial years of the Journal.
77
A Note of Thanks to Our Reviewers
Dale Abrahamson
Leona Aerts
Elif Arioglu
C.J. Bailey
Aydin Barlas
Hanoch Bar-On
T.K. Basu
Geert Jan Biessels
Kersten Buschard
Norman Cameron
Arthur Campfield
Richard Carr
Erol Cerasi
Tova Chajek
Subrata Chakrabarti
Mark E. Cooper
Mary Cotter
Errol D. Crook
N.S. Dhalla
R. Donnelly
Joseph C. Dunbar
Simon J. Dunmore
Shimon Efrat
Joseph Eichberg
George S. Eisenbarth
D.L. Eizirick
Robert B. Elliot
Eva Feldman
Peter Flatt
Josephine Forbes
Robert Frank
Jean Paul Giacobino
Per Henrik Groop
George Grunberger
Barbara Hansen
Leonard C. Harrison
William W. Hay
Bo Helman
Jean Claude Henquin
Douglas N. Henry
Andre Herchuelz
Emilio Herrera
David Hill
Kathleen Holemans
Thomas H. Hostetter
Gokhan S. Hotamisligil
Jorma Ilonen
Bowie Kahle
B. Kahn
Frederik Kaskel
Timothy Kern
George L. King
Iwar Klimes
Gregory Korbutt
Anjaneyulu Kowluru
Anna Krook
D. Langin
David Lau
Jack Leahy
Edward H. Leiter
Sigurd Lenzen
Ake Lernmark
Zecharia Madar
Errol Marliss
Michelle Marron
Clayton Mathews
Franz Matschinsky
Paolo Meda
Vincent Monnier
David Mott
Ram Nagaraj
Christopher Newgard
Irina Obrosova
Hiroshi Okamoto
Asher Ornoy
Jerry Palmer
Hans Parving
Ammon Peck
Michael Pfeifer
Masimo Pietropaolo
Andreas Plagemann
Bernard Portha
Guiseppe Pugliese
Alexander Rabinovitch
Ralph Rabkin
Arlene Ramsingh
Heimo Riedel
E. Ritz
Ladislas Robert
Jorge Tamarit Rodriguez
Bert Roep
Merja Roivainen
Guillermo Romero
Aldo Rossini
Dvora Rubinger
Ingo Rustenbeck
Stellan Sandler
Robert Schmidt
Fraser Scott
Assia Shisheva
Colin P. Sibley
Bhagirath Singh
Gail E. Sonnenshein
Robert L. Sorenson
Yechezkel Stein
Martin Stevens
Andrew Taylor
Markus Tiedge
Vincenzo Trischitta
Jerrold Turner
Thijen Utkan
Angela M. Valverde
L. Van Gaal
Pnina Vardi
Aaron Vinik
Ken Walder
Michael B. Wheeler
Morris White
John Wiley
Yagahashi, Soroku
Ji-Won Yoon
Mark Yorek
Daniel Ziegler
Juleen Zierath
Ehud Ziv
Anders A.F. Sima, Eleazar Shafrir
Editors-in-Chief

				

